# A STEM Course Analysis During COVID-19: A Comparison Study in Performance and Affective Domain of PSTs Between F2F and F2S Flipped Classroom

**DOI:** 10.3389/fpsyg.2021.669855

**Published:** 2021-08-10

**Authors:** Jin Su Jeong, David González-Gómez

**Affiliations:** Departamento de Didáctica de las Ciencias Experimentales y Matemáticas, Universidad de Extremadura, Cáceres, Spain

**Keywords:** STEM education, COVID-19, flipped classroom, F2F and F2S, e-learning, emotion and perception, performance

## Abstract

Due to the worldwide COVID-19 pandemic, university education has faced a significant challenge that requires adaptation to virtual and online education. Here, a fruitful flipped methodology with increased popularity can support adaption to and improvement of the current pandemic situation. This research presents a comparison of two different instruction situations with an identical teaching methodology, face-to-face (F2F) and face-to-screen (F2S) flipped methodology, in terms of students' performance and affective domain in a science, technology, engineering and mathematics (STEM) course. It was considered and designed as an examination of 132 pre-service teachers (PSTs), with 68 and 64 PSTs respectively for each group. The first group before the pandemic was applied by F2F flipped classroom and the second group after the pandemic was applied by F2S flipped classroom. The results after pertaining various data analyses of class activities and questionaries showed that performance had been improved for both groups toward the course. In addition, F2F had a significant difference in PSTs' emotion and perception toward the course and made classes more interactive. The mean score values of students' emotion and perception between two groups showed that the difference between these mean values were significant, suggesting a very large effect. Particularly, the effect size (ES) showed that positive emotions were more significant with different variables and the items Q7–Q9 of questionnaires indicated more significant different perceptions for both F2F and F2S after completing the course. Finally, the principal component analysis (PCA) test described that F2F answers were located mainly in the positive emotion, while F2S answers were grouped in the negative emotion, while no differences were observed for PSTs perceptions to the flipped methodology. Consequently, although F2F–F2S transition was an effective process, instructors and PSTs faced difficulties in the platform usage for online lectures reflecting emotions' results in F2S group. Thus, by solving the problems raised, it will allow PSTs to be more interactive in a virtual and online context for their future implementation by giving them active instruction methodology and educating future students to teach STEM contents.

## Introduction

Due to the COVID-19 pandemic, the United Nations Educational, Scientific, and Cultural Organization (UNESCO) indicated that educational institutions had been providing over 70% of their classes and assessments through various on-line and virtual platforms (Stub, 2020; UNESCO, 2020). Before the appearance of COVID-19, e-learning showed a consistent tendency of continuous growing, about 15.4% per year, in educational institutions (Wang et al., 2020). There was not any pressure or uncertainty for either institutions or students (Azeiteiro et al., 2015; Garg and Jain, 2017). However, learning environments were forced to change due to circumstances during the COVID-19 pandemic, which saw millions of cases confirmed in more than 216 countries according to the World Health Organization (WHO) (Cao et al., 2020; WHO, 2020a,b). The situation that began in the middle of the spring semester was totally unexpected and unplanned for by all people working in academic institutions, especially instructors and students. Due to precautious actions before the COVID-19 spread and lockdown, many institutions had already switched to complete e-learning teaching/learning (Cao et al., 2020; Crawford et al., 2020; WHO, 2020b). So, it resulted in a suspension of the current educational procedures and developments in many worldwide institutions (Fauci et al., 2020; WHO, 2020a). The traditional instruction method was changed to an e-learning model that allowed students to continue and finish their classes and activities (Crawford et al., 2020). Each institute adopted different and various e-learning systems due to the action of social distance regulation and directives, which the WHO strongly recommended to halt the COVID-19 spread amongst persons and countries (WHO, 2020a,b). Previous studies have indicated that instructors and students in educational areas had a close relationship and improvements in the on-line and virtual system (Moura et al., 2010; Azeiteiro et al., 2014; Islas-Pérez et al., 2015). However, this new and unfamiliar home-based education system was implemented to foster instructors and students to have a considerable responsibility of on-line and virtual skills and experiences (Bacelar-Nicolau et al., 2009; Crawford et al., 2020). Despite the enormous efforts to solve the glitches among the different teaching strategies implemented, the efficiency of on-line and virtual teaching was still falling behind expectations (Zare et al., 2016; Yang et al., 2017). Even though the instructors had undergone the training required in a brief period, there was a requirement to change the existing and paper-based materials to on-line and virtual teaching resources (Parkes et al., 2014; Cao et al., 2020). Therefore, it would be necessary to find out a proper methodology and system, which could achieve its objectives in education accomplishing the WHO social distancing suggestion during the COVID-19 pandemic.

In a teaching-learning process, especially considering current situations, on-line and virtual learning can be considered as a proper educational model (Eneroth, 2000; Paechter et al., 2010). This model, along with the information and communication technologies (ICTs), allows flexible and relevant student-focused education in science, technology, engineering, and mathematics (STEM) education (Pereira et al., 2008; Shee and Wang, 2008; Chujitarom and Piriyasurawong, 2019). ICTs can provide scaffolding among teacher to teacher, teacher to student, and student to student interactions that can help on-line and virtual systems as a virtual teaching-learning platform and multi-faceted communications (Garrison, 2000; Narciss et al., 2007; Pereira et al., 2008). The on-line and virtual system has various advantages that can be used without the consideration of time and place and can require a self-regulated learning practice (Garrison, 2000; Lee and Lee, 2008; Lozano et al., 2013). In teaching-learning forming fundamental factors for STEM education, it can be considered as inter- and multi-disciplinary development (Narciss et al., 2007; Lee and Lee, 2008; Lozano et al., 2013). According to Hansen (2001), the students in an on-line and virtual system typically had a greater sense of comprehension and experience, which led to fruitful transformative teaching-learning (Arbaugh, 2000; Schramm et al., 2001). Also, the students' achievements for learning as Paechter et al. (2010) indicated were closely connected to the on-line and virtual systems' characteristics, which were multi-directional communications for learning strategy flexibility and experience transmission, related with all significant issues of STEM education (Narciss et al., 2007; Moura et al., 2010; Lambrechts et al., 2018). On the other hand, these innovative systems proposed could be of great help for STEM education development in long-term teaching-learning (Garrison, 2000; Azeiteiro et al., 2014). Here, the technological integration to STEM education could fill a current educational niche, although there are many existing challenges, which will be integrated to transformational STEM teaching-learning (Pavlova, 2013; McVey, 2016; Nowotny et al., 2018). However, general on-line and virtual learning systems are still required to examine more specific models' efficiency in-depth, such as higher STEM education through e-learning systems (McVey, 2016; Nowotny et al., 2018). Thus, active methodologies based on on-line and virtual system are necessary to achieve their objectives in adopting STEM education during COVID-19.

The flipped classroom methodology, a form of active education methodology, recently gained a great level of attention in higher education along with the STEM courses (Roach, 2014; Blair et al., 2016; Ye et al., 2018). This methodology can provide a more suitable teaching-learning environment to reach significant and fruitful achievements together with the great availability of digital materials that comply with the WHO social distancing suggestion during the COVID-19 pandemic (Roach, 2014; Blair et al., 2016; Cao et al., 2020). In the view of students, O'Flaherty and Phillips (2015) indicated that this methodology required them to take responsibility for their learning. Sams and Bergmann (2013) mentioned that a flipped classroom course would be effective not only for a big group of students but also for individual students, unlike a traditional direct lecture. Particularly, in higher STEM education, traditional teaching methodology was better suited as an instructor-centered methodology to be delivered to students (Williams et al., 2018; Jeong and González-Gómez, 2020a). Here, the flipped classroom methodology aforementioned can be an effective and alternative approach delivering a student-centered methodology (Dooley et al., 2018; Zamora-Polo et al., 2019; Jeong and González-Gómez, 2021). In a basic flipped course setting, students can receive their lectures at home in the format of videos, tasks, quizzes, and written materials in on-line spaces such as Moodle. Reversely, students can do class activities that are conventionally done at home with instructors' supervision (Jeong and González-Gómez, 2020b). Here, more student-centered activities can be performed in-class time along with providing just-in-time lectures and collaborative tasks, which can address detailed questions, and realize more efficient chances for learning (Mattis, 2015; Moraros et al., 2015; Namaziandost and Çakmak, 2020). For this research, the performance in the flipped classroom methodology can be improved in the context of students' learning due to there being more in-class time along with active learning (Akçayir and Akçayir, 2018; Jeong and González-Gómez, 2020a). Kemp and Grieve (2014) indicated in their study that similar academic performance was achieved in both an on-line and face-to-face learning environment, but a higher preference of face-to-face settings was observed when students-centered activities were carried out. Although learning and affective domains could be considered as interdependent, the affective domains' influence on learning should be theoretically analyzed and practically investigated (Bower, 1981; Schwarz, 1990; Abele, 1995; Hascher and Edlinger, 2009). The learning process is the outcome of the cognitive and affective domain interplay (Pintrich et al., 1993). Currently, several theories show at least a certain amount of empirical evidence. Bower (1981) indicated a theory, “mood-congruence-hypothesis,” that information can be more easily remembered in a positive mood than in a negative mood. Thus, Schwarz (1990) suggested the theory “mood as information,” in which the pertainable point is the role that mood itself plays for learners. STEM education research was prevailing in the cognitive aspects of teaching/learning procedures without attention on the affective domain (Mellado et al., 2014). However, a growing interest has been seen on understanding the influence of emotions in the teaching/learning process by many studies in recent years (Dos Santos and Mortimer, 2003; Zembylas, 2007; Abrahams, 2009; Ritchie et al., 2011; Schutz and Zembylas, 2011; Bellocchi et al., 2013). Owing to support and higher interest in affective domain, students' perception can be analyzed for their opinions of flipped classroom methodology (Blair et al., 2016; Akçayir and Akçayir, 2018). Many studies have been carried out in different educational levels that have measured the students' perceptions toward the flipped classroom methodology (Bishop and Verleger, 2013; Roach, 2014; Gilboy et al., 2015; Sowa and Thorsen, 2015; Long et al., 2016). Abele (1995) demonstrated a positive mood can even increase the pace of perception along with performance and processing. Jeong et al. (2019) showed that perception and emotion had a significant relationship to students' learning in various learning environments in a STEM course, including face-to-face and face-to-screen learning settings, particularly the confirmation of more face-to-face education with different researchers (Marshall, 2011; Baker, 2012; Blair et al., 2016; Williams et al., 2018; Ye et al., 2018). The instruction methodology should also encourage students' positive perceptions and emotions toward STEM, especially in pre-service teachers (Osborne et al., 2003; Jarvis and Pell, 2004), who will be future instructors after their development and formation. Hence, based on the previous literatures and reasons confirmed, an active flipped methodology can be evaluated in different situations, face-to-face (F2F) and face-to-screen (F2S) flipped methodology in a STEM course, due to COVID-19, in terms of the students' performance and affective domains.

In this work, a comparison of two different instruction situations along with an identical teaching methodology is presented: F2F and F2S flipped methodology in terms of PSTs' performance and emotion and perception in a STEM course. A total of 132 PSTs participated in this study across two different years, 2018/19 and 2019/2020 course (68 and 64 PSTs, respectively). Students were randomly assigned to the studied group and they agreed to participated in this study. Particularly, before and after the COVID-19 pandemic, the first group was applied to the F2F flipped classroom and second group was applied to the F2S flipped classroom. With the various data analyses of class activities and questionaries, the results expose the performance variation and the significant change of emotion and perception of PSTs toward the course implemented. Also, it can show their effect size (ES) and principal component analysis (PCA) differences based on PSTs' data that allow PSTs to be more interactive and adopted in different instruction contexts.

## Materials and Methods

A flipped classroom instruction methodology was applied in a STEM class during two different courses with an identical instruction methodology before and after the COVID-19 pandemic. Precisely, a F2F flipped instruction methodology was followed in the first course and F2S flipped instruction methodology in the second one. The class used to study this methodology had a course syllabus containing overall themes of science along with the didactic method and strategies to teach these contents for primary education. For each course, PSTs were randomly assigned and agreed to participate in this research.

### Sample

For the course proposed, a total of 141 PSTs from two groups enrolled for this course. The PSTs were randomly assigned into individual courses, 70 and 71 PSTs, respectively. Here, the PSTs before registering for the subject did not have any knowledge of the flipped methodology for the course and choices were not based on any preconceived prejudices. The instructors imposed a constraint that there must be a similar quantity of participants for both groups. Each group had an identical instruction methodology in two different environments due to COVID-19: F2F flipped instruction methodology and F2S flipped instruction methodology, correspondingly. There were some PSTs who did not participate actively, which indicated the final response rate was 68 PSTs (97.14%) for F2F group and 64 PSTs (90.14%) for F2S group. The final participation rate was 132 PSTs (93.62%) for both groups, which was representative of the entire course. As displayed in Table 1, descriptive demographic information of PSTs showed a total participant number, gender distribution, average age, pre-test grade point average (GPA) that the average grade student had before starting the course, and educational background with social sciences, sciences, technology, arts, and others. Particularly, their average age was 20.1 and 21.0, respectively, with the total average age being 20.55 years old. In the case of gender distribution, it was a different pattern for each group. The pre-test GPA was 6.93 and 6.91 in the 0–10 scale, respectively. Finally, regarding the PSTs' educational background, both groups had a similar percentage, such as 68.3 and 65%, which showed the majority of PSTs did not have a strong STEM background from their previous education.

**Table 1:** The comparison of demographic background information between PSTs participating in the F2F and F2S flipped classroom study.

**Items**		**F2F Group**	**F2S Group**
Number of PSTs		68	64
Average age		20.1	21.0
Gender	Male	35.1%	54.0%
	Female	64.9%	46.0%
Pre-test GPA (Maximum 10)		6.93	6.91
Educational background	Social sciences	68.3%	65%
	Sciences	14.5%	20%
	Technology	1.8%	0%
	Arts	5.1%	5%
	Others	10.3%	10%

### Instructional Design

This study was conducted in a general STEM course across two different years. The STEM course is called “Teaching of Matter and Energy” and is taught to the PSTs as a mandatory subject. The course consists of 3 h per week on theoretical contents and 1 h per week for laboratorial contents. Particularly, in the theoretical classes, all students attend simultaneously, in the same classroom for the F2F or remotely from their homes for the F2S group. In experimental contents, the class is divided into three groups, which allows for the provision of better instruction for the laboratorial activities. Together with the theoretical and laboratory class hours, the PSTs can arrange an individualized tutorial period with the instructors to clarify contents. In both F2F and F2S courses, the same syllabus was followed, and the course had the same structure. As is shown in Table 2, the course structure consists of five units including overall features and views of the matter and the energy. In this methodology in two different environments, an active and participatory atmosphere was promoted for their learning.

**Table 2:** The general information of the subject implemented into the course for F2F and F2S flipped classroom.

**Chapter**	**Title**	**Description**
1	Science teaching/learning in primary education	19 h: This chapter consists of scientific literacy, primary science education, teaching models, strategies, techniques, and resources to instruct science in primary education.
2	The Universe	33.5 h: This chapter consists of the Universe's origin and evolution, the fundamental structure of the Universe, the solar system, the Sun, the Earth, the Moon, and the model of Sun-Earth-Moon for primary education.
3	Matter	32 h: This chapter consists of matter's physical and chemical properties with its interactions, atomic models, substances/mixtures, density, and mechanics/fluid mechanics.
4	Matter transformation	33.5 h: This chapter consists of physical changes, thermodynamics, chemical changes/reactions, and nuclear changes/reactions.
5	Energy	32 h: This chapter consists of energy types, energy transformation/transfer, conservation and degradation, energy use, light/sound, electric energy (circuits/magnetism), and energy, society, and environment.
	Total	150 h

**Figure 1:**
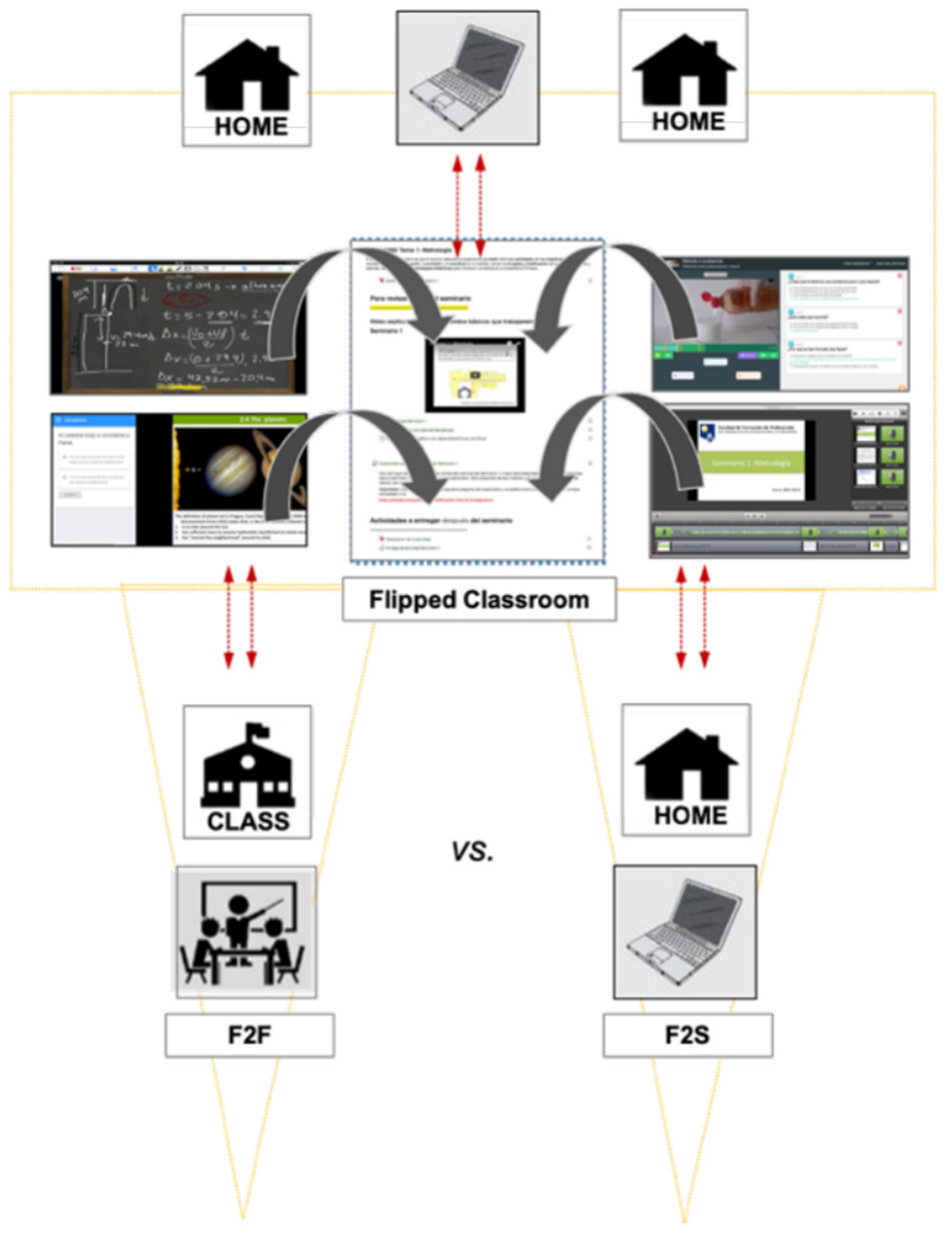
A conceptual framework of a STEM course to examine performance and affective domain between F2F and F2S flipped classroom.

In both F2F and F2S courses, the flipped paradigm was presented from the beginning of the course and all PSTs had access to the virtual interface of Moodle that contains the course flowchart with all the principal dates and subject activities scheduled. In both cases, the flipped material consisted of pre-recorded video-lessons and lab demonstrations and texts that PSTs received, based on the syllabus, 1 week before working on them synchronously (F2F and F2S). So, PSTs can prepare for the class while watching the flipped materials. Here, they can retrieve all of the material for the whole course. Particularly, PSTs also had access to an online quiz based on a multiple-choice format that examined the subject contents. Then, they can give feedback to the instructors before the actual class, which can be considered as a “just-in-time” lecture if necessary. In this active class environment, PSTs can use in-class time to engage more with the class activities than just passively participating in the class. Figure 1 displays a class session structure for F2F and F2S flipped instruction methodology, which incorporates a schematic vision of two groups' learning processes.

### Comparison of Students' Emotions and Perceptions Toward F2F and F2S

The comparison study between the same instruction methodology in two different environments was realized by a questionnaire survey that PSTs completed at the end of the course. The questionnaire was formed on the basis of previously published research by Roach (2014) and then was adapted to the current work after considering the course syllabus and contents. Thus, an expert panel of professors and researchers working in this topic along with the university bioethics board validated the questionnaire before collecting the information.

The designed questionnaire consisted of three different sections. The first section (section 1 Introduction) was for gathering PSTs' demographic information such as age, gender, current GPA, and educational background (see Table 1). The second one (section Materials and Methods) was for collecting the data of PSTs' emotions when F2F and F2S flipped instructional methodology were followed. Here, emotions were divided into two groups, positive and negative emotions (Bisquerra, 2005). Fun, confidence, enthusiasm, and tranquility were used for the positive emotions and nervousness, concern, boredom, and fear were used for the negative emotions (Dunbar et al., 2016). PSTs answered their opinions based on the 0–10 scale about their emotions assessed toward the course, with the lowest incidence indicating 0 and the highest incidence indicating 10.

As shown in Table 3, the third section (section Results and Discussion) was dedicated to collecting the data of PSTs' perceptions when F2F and F2S flipped instructional methodology was followed. Here, the questionnaire consisted of nine questions that were closed type and could be defined and arranged in two groups. The first group consisted of six questions Q1 to Q6, which inquired about flipped video and other activity materials' suitability and how these flipped materials were valuable to achieving the learning and proficiencies of course goals. The second group consisted of three questions Q7 to Q9, which inquired about the entire flipped classroom and the valuable studying experience of the course (Roach, 2014; González-Gómez et al., 2019). Also, PSTs answered their opinions based on 1–5 as a five-point Likert-type scale about their perceptions assessed toward the course, which ranged from strongly disagreed (SD), disagreed (D), neutral (N), agreed (A), and strongly agreed (SA).

**Table 3:** Five-points Likert-type survey used in this study to compare the F2F and F2S in terms of PST's perception change (Section Results and discussion).

**Group of questions**	**Question**	**Description**
1	Q1	I would take another course that used the same scheme as the one followed in this study.
	Q2	The video lectures helped me to learn.
	Q3	Watching the video lectures and revising the provided materials before the class sessions helped me to complete the in-class activities in a more confident manner.
	Q4	Watching the video lectures and revising the provided materials before the lab sessions helped me to easily complete the proposed activities.
	Q5	The completion of multiple-choice on-line quizzes after watching the delivered video lectures allowed me to point out the most complex contents before the class, and therefore focus on overcoming them.
	Q6	Discussing with classmates and other collaborative activities helped me to learn.
2	Q7	The course as a whole was a valuable learning experience.
	Q8	The course was more interactive when compared with others.
	Q9	The instruction methodology used in this course will be useful to apply in other subjects.

### Statistical Analysis

The gathered data throughout the instruments implemented were analyzed in a quantitative manner. Firstly, a descriptive analysis was used to represent the sample data conclusions as the most suitable method to describe, characterize, and draw (Etxeberria and Tejedor, 2005; Jeong et al., 2019). Then, a Cronbach alpha test was used to check the reliability of questionnaires (Pintrich and De Groot, 1990; Biggs et al., 2001; Ahlfeldt et al., 2005). Table 4 shows the value of Cronbach Alpha test for the two divisions, emotion and perception, and indicates emotion's question validity was 0.89 and the perceptions' question validity was 0.93. Consequently, for both questionnaire sections, the Cronbach alpha test can be determined as acceptable as it is close to reliable when making an important decision (Biggs et al., 2001).

**Table 4:** Cronbach Alpha test in this study for the questionnaire Sections Materials and Methods and Results and Discussion.

**Variables**	**Questions**	**Cronbach Alpha value**
Emotion (Section Materials and Methods)	8	0.89
Perception (Section Results and Discussion)	9	0.93

The Kolmogorov–Smirnov test for normality was used to check whether data collected were normally distributed or not. Here, the data gathered were normally distributed, so we conducted a parametric statistical test. To find a significant difference and relationship between data of F2F and F2S, *t*-test as a parametric statistical analysis was performed at 95% confidence level. Both emotion and perception data were examined by score mean values that were compared and showed the significant differences' presence by means of *t* test at 95% confidence level. Then, the effect size (ES) estimation was executed in accordance with the Rosenthal method (Rosenthal, 1991). According to Cohen (1988), the ES was applied to gauge the treatment effect extent. Finally, principal component analysis (PCA) was used to deduce whether all data gathered had an objective to conduct. As a useful tool, the PCA can summarize large quantities of data. Also, it can conclude how samples collected are different from each other (F2F and F2S data), how variables can serve more significantly to the variance, and how variables can correlate with each other (Peres-Neto et al., 2005; González-Gómez et al., 2019). Finally, SPSS statistics 22.0 software was used to find out all information.

## Results and Discussions

Through the different environments with an identical flipped methodology, the results obtained showed various examinations together with performance, emotion, and perception comparison. Particularly and firstly, sample homogeneity and performance comparison were checked to complete the comparative manner of this work. Then, the comparison of emotion and perception analysis was accomplished to figure out a keener vision of PSTs' affective domain. Consequently, the results showed the principal patterns and outlines for directing performance and affective domain analysis of a STEM course during COVID-19 with a comparison study between F2F and F2S flipped classroom.

### Sample Homogeneity and Performance Comparison

Table 1 describes the interesting aspect of a sample that nearly three-fourths of PSTs during the mid- and high-school stage did not take science subjects. Particularly, 20.1% of the F2F group and 15.1% of the F2S group did take science subjects during mid- and high-school stage. So, a high percentage of PSTs already lacked an understanding of the fundamental science concepts that would create many difficulties in understanding the subject. In order to finish the comparative study, the sample homogeneity if normally distributed or not was proven with reference to F2F's and F2S's emotions and perceptions. Here, the significant differences were detected between them.

In accordance with the university's statistical data stipulated during the previous 10 years, PSTs with a science background or not always had complications to finish the subject compared with the entire degree program. In reality, many PSTs took 2.5 years on average to finish this subject. Furthermore, there was an even smaller number of PSTs who took more than 4 years to finish this subject satisfactorily. Here, the performance results to gauge the proposed methodology success were compared between F2F and F2S groups. Table 5 summarized the results gotten for F2F and F2S teaching methodology. The final grade for each group shows 7.54 (F2F) and 7.23 (F2S), respectively. For flipped instruction methodology of F2F and F2S, even though the final pass rate of PSTs increased enough (67.1 and 70.3%), around 30% of students still needed to take the course again. Finally, the information about the pass rate in the two attempts that the university provided is also summarized in Table 5. Although for both groups, the pass rate were similar, higher scores were observed for the F2S groups, although no significant differences were established.

**Table 5:** Performance comparison between F2F and F2S.

**Teaching method**	**Number of PSTs enrolled**	**Number of PSTs participating in the study**	**Pass rate of 1st attempt of PSTs**	**Pass rate of 2nd attempt of PSTs**	**Pass rate percentage**
F2F	70	68	31	47 (31 + 16)	67.1%
F2S	71	64	33	51 (33 + 18)	70.3%

### Course Emotions Comparison

**Figure 2:**
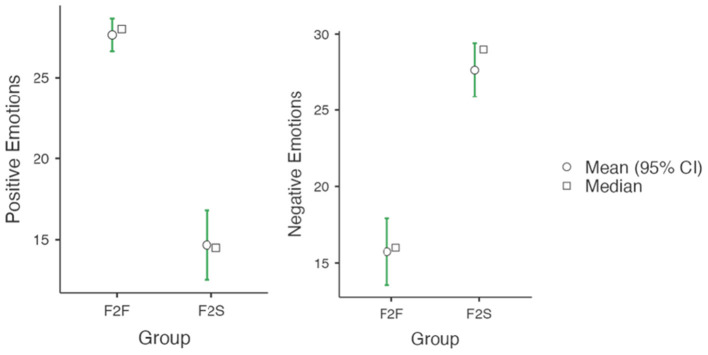
Emotion analysis for F2F and F2S flipped classroom after the course completion.

Figure 2 summarized the PSTs' comparison of emotions toward the flipped instruction methodology following F2F and F2S after finalizing the course. All scores of positive and negative emotions based on the statistical comparison analysis were significantly different in both F2F and F2S instructional settings. The mean score values for positive emotions for the F2F group was 27.6 (std dev = 4.24), whereas the mean score value for the F2S group was 14.7 (std dev = 8.71). The *t*-test showed that the difference between these mean values were significant (*p*<*0.001, d* = *1.91*), suggesting a very large effect. On the other hand, the score values for the negative emotions for the F2F group was 15.7 (std dev = 9.17), while the mean score value for the F2S group was 29.0 (std dev = 7.19). Again, the *t*-test showed that the difference between the mean values of both groups were significant (*p*<*0.001, d* = 1.44), which corresponded with a very large effect.

**Figure 3:**
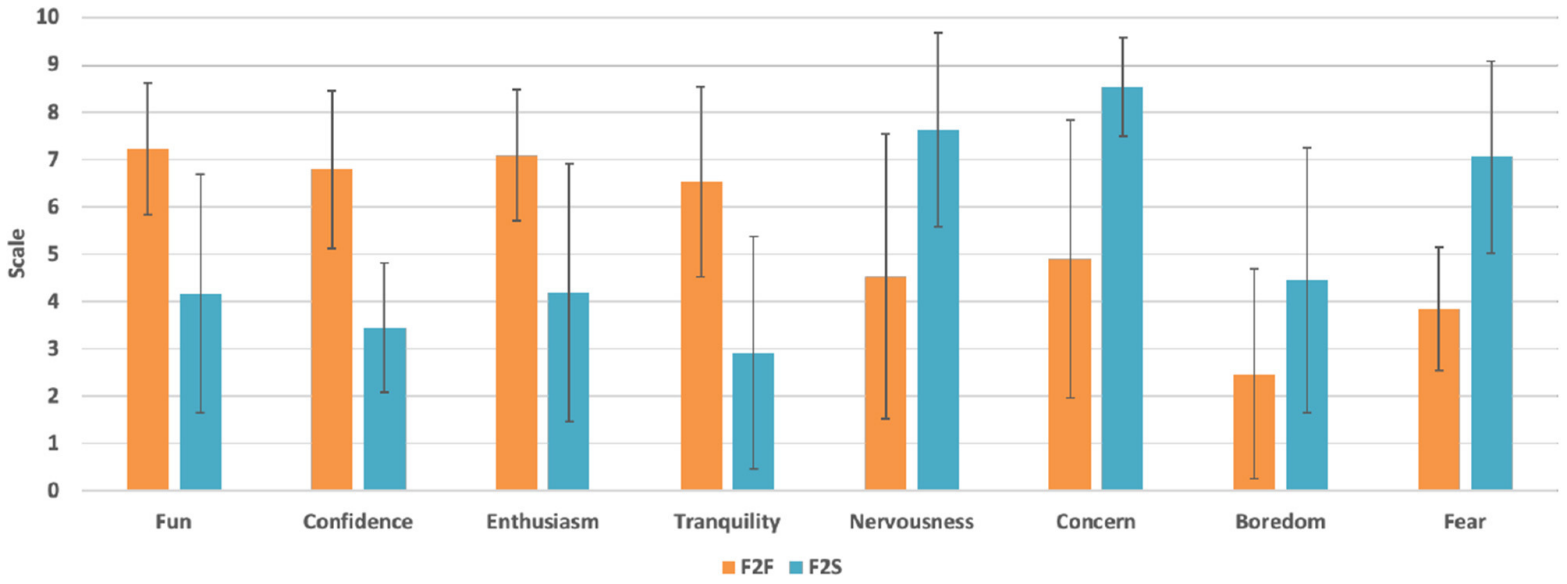
Single item assessment of the emotions for F2F and F2S flipped classroom after the course completion.

To assess the difference among emotions, each one of the assessed emotions were analyzed. The main results are summarized in Figure 3. Thus, with respect to the positive emotions, the F2F group showed high scores that had an average 3.1 points difference of the points specified by the F2S PSTs. Particularly, the positive tranquility emotion had a notable score, which had more difference among the positive emotions (6.53 in F2F and 2.92 in F2S course). On the contrary, F2S had a high score of negative emotions, with the negative concern emotion indicating more difference among the negative emotions (4.90 in F2F and 8.53 in F2S course). Also, the negative fear emotion along with negative concern emotion pointed out a big difference between F2F and F2S groups (3.84 and 7.05, respectively). The negative boredom emotion in both groups showed the lowest value, which was 3–4 points less than other negative emotions recognized.

Table 6 described the PSTs' comparison of emotions toward the flipped instruction methodology following F2F and F2S after finalizing the course. There are some scores of positive and negative emotions based on the statistical comparison analysis that were significantly different in both F2F and F2S instructional settings. Thus, the ES analysis specified that the instruction methodology had a medium to large effect in the whole emotion measured as indicated in the table (Cohen, 1988). Particularly, positive emotions' ES was all very large effects of significant different variables and negative emotions' ES was situated from medium to very large effect of significant different variables. Here, we have used the Cohen's *d* to find out the significant difference for each variable between two means divided by a standard deviation for the data as shown in Equation (1). The value of *d*'s magnitude indicates the ES size: very small is between 0 and 0.01, small is between 0.02 and 0.20, medium is between 0.21 and 0.50, large is between 0.51 and 0.80, very large is between 0.81 and 1.20, and huge is between 1.21 and 2.00.

(1) $$\begin{array}{l} s=\;\sqrt{\frac{\left(n_{1}-1\right)s_{1}^{2}+\left(n_{2}-1\right)s_{2}^{2}}{n_{1}+n_{2}-2}} \end{array}$$

**Table 6:** Emotion comparison between F2F and F2S flipped instruction methodology.

**Method**	**Fun**	**Confidence**	**Enthusiasm**	**Tranquility**	**Nervousness**	**Concern**	**Boredom**	**Fear**
F2F (SD)	7.23 (1.40)	6.79 (1.66)	7.09 (1.39)	6.53 (2.01)	4.53 (3.00)	4.9 (2.93)	2.47 (2.22)	3.84 (1.30)
F2S (SD)	4.17 (2.51)	3.45 (1.36)	4.19 (2.72)	2.92 (2.46)	7.63 (2.05)	8.53 (1.04)	4.45 (2.79)	7.05 (2.03)
*p*-values	0	0	0	0	0	0	0	0
ES (*d*)	1.52	1.65	1.34	1.61	1.13	1.54	0.78	0.97
	Very large	Very large	Very large	Very large	Large	Very large	Medium	Large

*All the assessed emotions were statistically different (parametric t-test at 0.05 significance level). The ES means of Rosenthal approach for the significant different variables*.

### Course Perceptions Comparison

**Figure 4:**
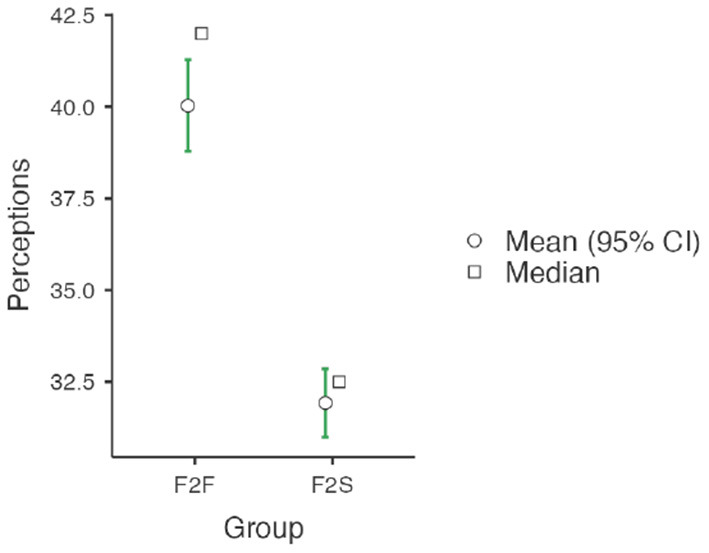
Perception analysis of F2F and F2S flipped classroom after the course completion.

Table 7 represented the PSTs' comparison of perceptions toward the flipped instruction methodology following F2F and F2S after finalizing the course. The main values of each questionnaire can be found in this table. Moreover, to catch a closer observation of PSTs' perceptions for both F2F and F2S instruction methodologies, Figure 4 summarizes the responses collected for the perception questionnaire part. The mean score values students' perception for F2F group was 40.0 (std dev = 5.26), whereas the mean score value for F2S group was 31.9 (std dev = 3.79). The *t*-test showed that the difference between these mean values were significant (*p*<*0.001, d* = *1.76*), suggesting a very large effect.

**Table 7:** Perception comparison between F2F and F2S flipped instruction methodology of the Likert-type test.

**Method**	**Group 1**						**Group 2**		
	**Q1**	**Q2**	**Q3**	**Q4**	**Q5**	**Q6**	**Q7**	**Q8**	**Q9**
F2F (SD)	4.50 (0.76)	4.52 (0.61)	4.47 (0.72)	4.37 (0.73)	4.43 (0.82)	4.50 (0.72)	4.28 (0.84)	4.47 (0.70)	4.50 (0.70)
F2S (SD)	3.89 (0.88)	4.09 (0.85)	4.33 (0.76)	4.28 (0.75)	3.41 (0.97)	3.58 (0.97)	3.03 (1.07)	2.56 (1.10)	2.75 (1.02)
*p*-values	0.000	0.000	No Sig.	No Sig.	0.000	0.000	0.000	0.000	0.000
ES (*d*)	0.69	0.51	0.17	0.10	1.08	1.01	1.25	2.03	1.93
	Medium	Medium	–	–	Large	Large	Very large	Huge	Very large

*All the assessed perception questionaries were statistically different (parametric t-test at 0.05 significance level). The ES means of Rosenthal approach for the significant different variables*.

Again, to have a detailed view of the perceptions, each item was also analyzed. According to the statistical comparison, Table 7 showed that some perception scores are significantly different in both F2F and F2S instructional environments. However, Q3 and Q4 questionnaires' scores indicated that there were significant differences in statistical assessment by both F2F and F2S groups. The rest of the questions for perception scores provided were significantly different in both F2F and F2S instructional environments. In addition, the ES analysis indicated that the instruction methodology had a small (no ES) to huge ES in the perception assessed as indicated in the table (Cohen, 1988). Particularly, Q1–Q6 perception items were located in between small (no ES) to large variables and the Q7–Q9 perception items were located in between very large to huge variables. Here, we used the same manner of Cohen's *d* to find out their significant difference for each variable between two means divided by a standard deviation for the data aforementioned.

**Figure 5:**
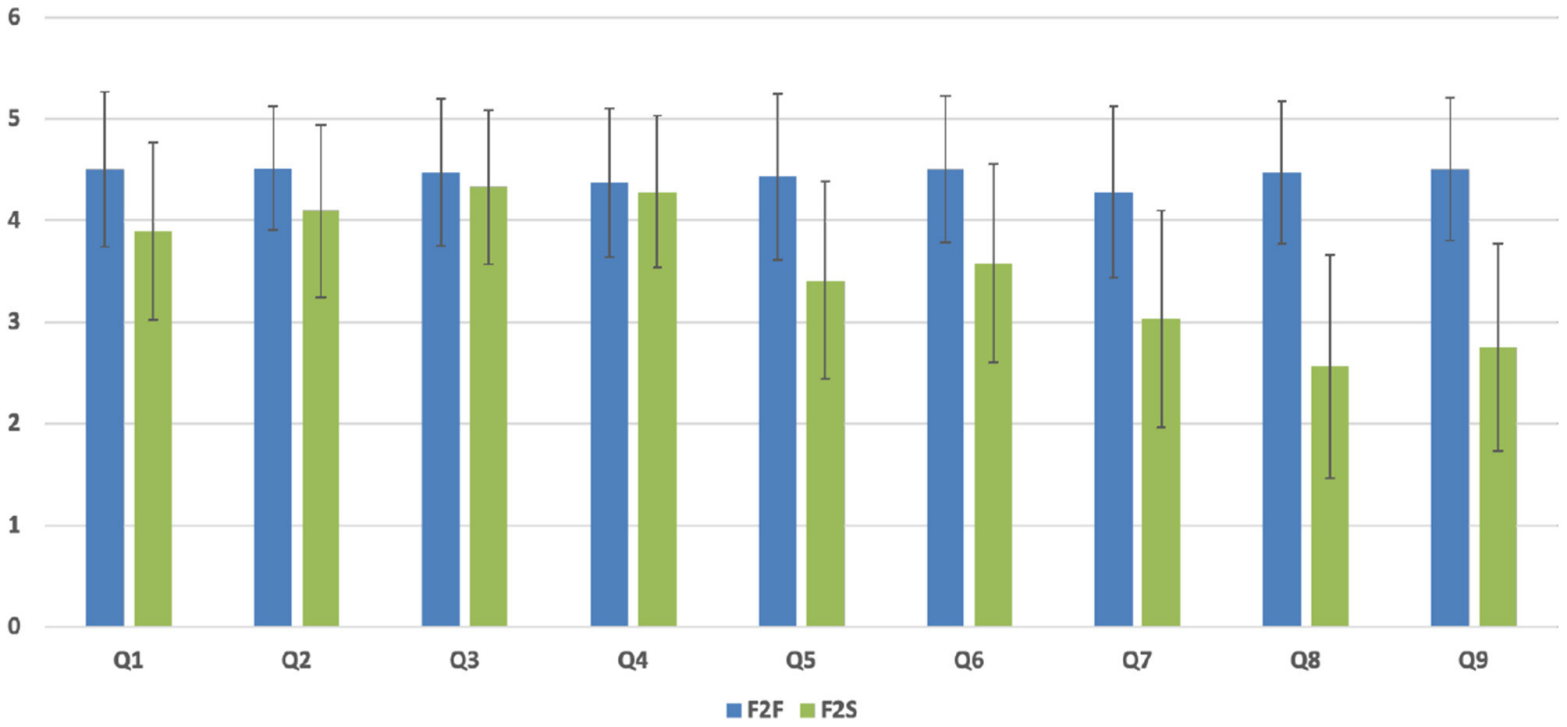
Single item assessment of the perceptions of F2F and F2S flipped classroom after the course completion.

Figure 5 showed the PSTs' perception part of the questionnaire with a closer view for both F2F and F2S instruction methodologies provided. According to the statistical comparison analysis, all perception questionnaires provided were significantly different in both instructional environments. Particularly, Q1, Q2, Q5, and Q6 items had around a 0.5 increase between F2F and F2S while Q3 and Q4 items had 0.14 and 0.19 difference, respectively, in the Group 1 questionnaires. In the Group 2 questionnaires, Q7–Q9 had more than 1 point and even close to 2 points difference between F2F and F2S groups after the course completion. Specifically, the Q8 item described PSTs' perceptions about the course learning experience as a whole. In this item, the average score of F2F was 4.47 points while the F2S showed 2.56 points. So, the F2F offered a higher positive perception about the learning procedure for the identical contents and could be considered as a significant contribution in PSTs' learning involvement and practice. Thus, both F2F and F2S groups agreed or strongly agreed the practicality of video lectures and other flipped materials before class improved learning target attainment and allowed PSTs to achieve the in-class works more confidently and easily. Particularly, those PSTs enrolled in the F2F course agreed or strongly agreed to have more flipped and presential course as the teaching methodology as a whole, which assisted in realizing their learning purposes.

### PCA Test

**Figure 6:**
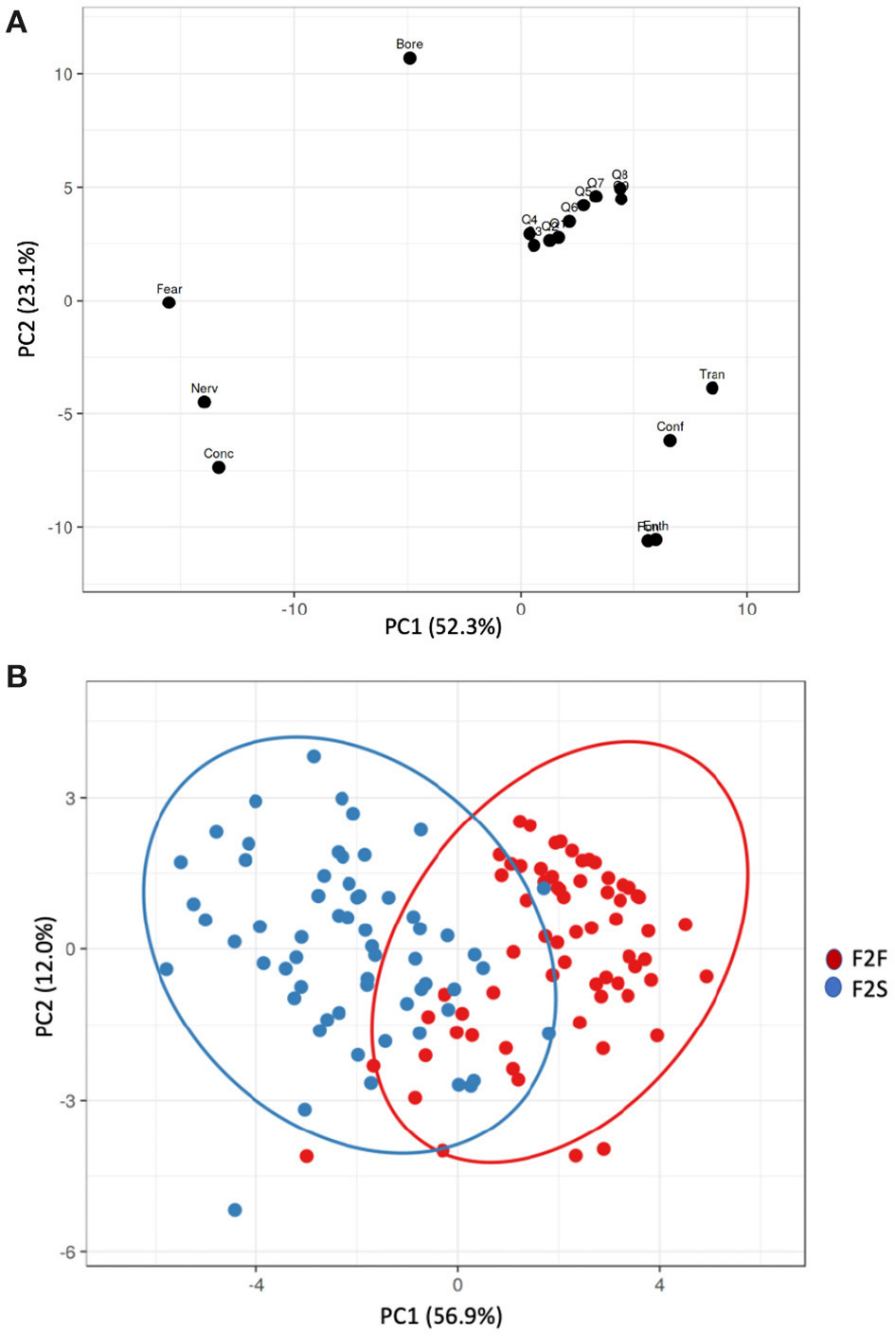
The PCA diagram: **(A)** loading corresponding to the questionnaires of emotion and perception; **(B)** scores plots corresponding to the PSTs distribution of F2F and F2S flipped classroom.

Figure 6 showed the PCA analysis in order to get a clear image of the instruction methodology's effect over the PSTs' emotion and perception toward STEM between F2F and F2S flipped instruction methodology. Here, it indicated a PCA loadings diagram about emotion and perception, in which the X and Y axes showed principal component 1 and principal component 2, that explained 52.3 and 23.1% of the total variance, respectively. According to the PCA results, the first PC was able to group the sample in to two groups (F2F and F2S answers). Precisely, prediction ellipses were also added to the PCA scores plot to show the probability that a new observation from the same group (F2F or F2S) will fall inside the ellipse with a 95% probability. Thus, F2F answers were located mainly in the positive axis of PC1, while F2S answers were grouped in the negative part of PC1. When comparing the score and loading plots, it is clear that PC1 represented the effect of the instruction methodology in the emotion toward science of PSTs, and it was able to distinguish between F2F and F2S groups. F2F scores were in the positive axis of PC1 which corresponds with positive emotion, while the F2S scores were in the negative axis of PC1 that corresponds with the negative emotions. Regarding the loadings corresponding to the students' perception, they are also located in the positive axis of PC1, and therefore are more correlated with the F2F scores.

### Discussion

The obtained outcomes show the information that can be considered as a novel approach to examine PSTs performance and affective domains of F2F and F2S flipped instruction methodology during the COVID-19 pandemic. This research stipulates an exclusive comparison for a specific flipped STEM education of PSTs and can fill a niche/gap of different environments with an identical methodology to measure performance and affective domains.

Although on-line and virtual learning showed a consistent growing tendency, many institutes were not prepared for a significant transition due to the COVID-19 pandemic (UNESCO, 2020; WHO, 2020a,b). Many researchers confirmed that on-line and virtual learning along with the ICTs could be considered as a proper educational model in a teaching-learning process of STEM course (Lee and Lee, 2008; Pereira et al., 2008; Shee and Wang, 2008). Here, among teacher to teacher, teacher to student, and student to student interactions, ICTs can act as a scaffolding for communications (Garrison, 2000; Lee and Lee, 2008; Lozano et al., 2013). Thus, the technological integration to STEM education could fill a current educational niche, although there are existing many challenges, which will be integrated to transformational STEM teaching-learning (Eneroth, 2000; Paechter et al., 2010; Nowotny et al., 2018). On the other hand, these innovative systems proposed could be of great help for STEM education development in long-term teaching-learning (Garrison, 2000; Azeiteiro et al., 2014). However, general on-line and virtual learning systems are still required to examine more specific models' efficiency in-depth such as higher STEM education through e-learning systems (McVey, 2016; Nowotny et al., 2018). Therefore, a proper methodology and system proposed could achieve its objectives adapting to STEM education while complying with COVID-19 guidelines.

Together with the confirmation aforementioned, the flipped classroom methodology along with digital materials in STEM can provide a more suitable teaching-learning environment to reach significant and fruitful achievements, while complying with the WHO social distancing suggestions during the COVID-19 pandemic (Blair et al., 2016; Cao et al., 2020; WHO, 2020a). Previous studies also found that video-lectures supported shaping conceptual understanding and was valuable the convenience of students (Imran, 2013; Roach, 2014; Long et al., 2016). The performance can be improved in the context of students' learning due to having more in-class time along with active learning integration and consequence (Akçayir and Akçayir, 2018). Pintrich and De Groot (1990) indicated that positive emotions were vital for promoting significant learning in the STEM course along with the theories of emotions and learnings analyzed and investigated by previous researchers (Bower, 1981; Schwarz, 1990; Abele, 1995; Hascher and Edlinger, 2009). Here, students' perception can be analyzed for their opinions of flipped classroom methodology (Blair et al., 2016; Akçayir and Akçayir, 2018). Kemp and Grieve (2014) also concluded that, although same academic performance was achieved in an F2F and F2S environment, students do prefer to accomplish specific activities in F2F settings. Thus, in the context of affective domain, Marshall (2011) indicated a significant relationship of students' learning favoring to F2F along with previous research in F2F and F2S setting (Baker, 2012; Blair et al., 2016; Jeong et al., 2019). Hence, based on the previous literature and reasons confirmed, a proper methodology and system proposed could achieve its objectives with the comparison of face-to-face (F2F) and face-to-screen (F2S) flipped methodology. Thus, it can overcome the COVID-19 situation in terms of the students' performance and affective domains.

Consequently, although the transition from the F2F to F2S classes could be considered as a positive process in institutes, the instructors and students confronted struggles and difficulties in the platform use for on-line and virtual classes. Along with these problems, the performance and affective domain results obtained by the PSTs specified the direction to follow; there were crucial considerations that future teachers were required to reflect on their on-line and virtual classes along with improvements from these instructions to better equip themselves for future classes. Particular attention was required in students' emotions during on-line and virtual class when instructors engaged. Also, specific comparison results obtained with the methodologies and objectives in the PSTs performance and affective domain of flipped STEM education could be reapplied to various educational areas and contexts when there were available data required due to its flexible characteristics.

## Conclusions

The research shows an examination of two different situations' comparison of an identical flipped instruction methodology, F2F and F2S, in terms of students' performance, emotion, and perception in a STEM course before and after the COVID-19 pandemic. It was designed and considered as a randomization examination with 132 PSTs, 68 and 64 PSTs, for the primary education bachelor's degree in Spain. Here, various statistical analyses were applied to data and questionnaires proposed.

According to the results obtained in this study, both groups of PSTs' increased their grade without significant difference after the course completion. The F2F had a significant effect on PSTs' perception and emotion toward the course and created classes that were more interactive. Particularly, in the comparison of students' emotions toward the instruction methodology, the ES analysis indicated that positive emotions' ES was all very large for significant different variables and negative emotions' ES was situated from medium to very large for significant different variables for both F2F and F2S after completing the course. Then, in the comparison of students' perceptions toward the instruction methodology, section 3 of the questionnaire (items Q7 to Q9) indicated a significant difference as ES showed a very large to huge index after the course was completed for both groups of F2F and F2S. Finally, the PCA test described that F2F answers were located mainly in the positive emotion part of PC1, while F2S answers were grouped in the negative axis of PC1. The scores plots indicated that positive emotions and perceptions were located in the positive axis of PC1 while negative emotions were grouped in the negative axis of PC1.

Consequently, although the conversion from F2F–F2S in the higher education was a fruitful procedure, students and instructors confronted difficulties in the use of online classes through a platform. In this research, the comparison demonstrated how to reflect the challenges and drawbacks meaningfully by the results obtained, especially emotions in the F2S group. Here, they emphasized emotions as the crucial criteria that instructors needed to consider when teaching virtual and online classes and taking advantage of these findings to better equip themselves for future classes. Although the F2F flipped classroom has enough virtual and online content, for the F2S classroom, more appropriate adoption and transition is required to promote both performance and affective domains of PSTs. Thus, it will allow PSTs to be more interactive in virtual and online context for their future implementation with active instruction methodology to educate future students to teach STEM contents. Finally, the main limitations of this study could be found in the sample size and the lack of analysis of other variables that might influence the results, such as participants' gender.

## Data Availability Statement

The raw data supporting the conclusions of this article will be made available by the authors, without undue reservation.

## Ethics Statement

The studies involving human participants were reviewed and approved by La Comisión de Bioética y Bioseguridad de la Universidad de Extremadura, Spain. The patients/participants provided their written informed consent to participate in this study.

## Author Contributions

All authors listed have made a substantial, direct and intellectual contribution to the work, and approved it for publication.

## Conflict of Interest

The authors declare that the research was conducted in the absence of any commercial or financial relationships that could be construed as a potential conflict of interest.

## Publisher's Note

All claims expressed in this article are solely those of the authors and do not necessarily represent those of their affiliated organizations, or those of the publisher, the editors and the reviewers. Any product that may be evaluated in this article, or claim that may be made by its manufacturer, is not guaranteed or endorsed by the publisher.
